# Interplay between IDO1 and iNOS in human retinal pigment epithelial cells

**DOI:** 10.1007/s00430-019-00627-4

**Published:** 2019-07-02

**Authors:** Katrin Spekker-Bosker, Christoph-Martin Ufermann, Maike Oldenburg, Walter Däubener, Silvia Kathrin Eller

**Affiliations:** Institute of Medical Microbiology and Hospital Hygiene, Heinrich-Heine-University Düsseldorf, Universitätsstr. 1, Bldg. 22.21, 40225 Düsseldorf, Germany

**Keywords:** Endogenous bacterial endophthalmitis, Indoleamine 2,3-dioxygenase-1, IDO1, Inducible nitric oxide synthase, iNOS, Retinal pigment epithelial cells, *Staphylococcus aureus*, *Toxoplasma gondii*

## Abstract

Human retinal pigment epithelial (hRPE) cells form a selectively permeable monolayer between the neural retina and the highly permeable choroidal vessels. Thus, hRPE cells bear important regulatory functions and are potential targets of pathogens in vivo. Endogenous bacterial endophthalmitis (EBE) is frequently caused by infections with the Gram-positive bacterium *Staphylococcus aureus (S. aureus)*. Upon microbial infection, interferon gamma (IFN-γ), a major cytokine of the adaptive immune response, induces a broad spectrum of effector molecules, such as the tryptophan-degrading enzyme indoleamine 2,3-dioxygenase-1 (IDO1). We stimulated human RPE (hRPE) cells in vitro with proinflammatory cytokines and analyzed the expression levels and enzymatic activities of IDO1 and inducible nitric oxide synthase (iNOS), another antimicrobial effector molecule. The antimicrobial capacity was analyzed in infection experiments using *S. aureus* and *Toxoplasma gondii* (*T. gondii*). Our aim was to characterize the particular importance of IDO1 and iNOS during EBE. We found that an IFN-γ stimulation of hPRE cells induced the expression of IDO1, which inhibited the growth of *T. gondii* and *S. aureus*. A co-stimulation with IFN-γ, interleukin-1 beta, and tumor necrosis factor alpha induced a strong expression of iNOS. The iNOS-derived nitric oxide production was dependent on cell-culture conditions; however, it could not cause antimicrobial effects. iNOS did not act synergistically with IDO1. Instead, iNOS activity inhibited IDO1-mediated tryptophan degradation and bacteriostasis. This effect was reversible by the addition of the iNOS inhibitor *N*^G^-monomethyl-l-arginine. In conclusion, iNOS mediates anti-inflammatory effects in hRPE cells stimulated with high amounts of IFN-γ together with tumor necrosis factor alpha and Interleukin-1 beta and prevents potential IDO1-dependent tissue damage.

**Electronic supplementary material:**

The online version of this article (10.1007/s00430-019-00627-4) contains supplementary material, which is available to authorized users.

## Introduction

Endophthalmitis and infectious retinitis are sight-threatening diseases, which are characterized by infections and subsequent proinflammatory immune responses. The strict control of proinflammatory responses is required to minimize tissue damage [1, 2]. The immune response against pathogens is dependent on the production of interferon gamma (IFN-γ), which is mainly derived from NK cells and T cells [3]. However, the eye is an immune-privileged organ and the vulnerable neurosensory cells have to be protected from damage by proinflammatory effector cells. Retinal pigment epithelial (RPE) cells are able to regulate T cell responses in the eye [2, 4] and are well known effector cells against *Toxoplasma gondii* (*T. gondii*) [5, 6] and cytomegalovirus (CMV) [7, 8]. Both cause severe diseases in immunocompromised patients, for example, retinitis, pneumonia, and encephalitis.

An exogenous endophthalmitis frequently occurs after penetrating ocular traumata and is a serious complication after eye surgery. In this case, environmental pathogens such as bacteria or fungi invade the eye and cause an infection. Despite the fact that exogenous endophthalmitis is a harmful infection, it is usually no source of bacteremia [1]. Post-surgery infections mostly occur due to coagulase negative staphylococci and propionibacteria, while the majority of post-traumatic exogenous endophthalmitis cases are caused by *Bacillus cereus* [1].

In contrast to an exogenous endophthalmitis, endogenous bacterial endophthalmitis (EBE) is a rare infection, but is often associated with poor visual outcome and can be life threatening. EBE is usually a complication of a systemic infection. During a systemic infection, characterized by sepsis (positive blood culture in 58%) and fever (in 74%), bacteria are distributed via the blood stream throughout the body. Bacteria that reach the eye can cross the blood–retinal barrier and establish EBE. *Staphylococcus aureus* (*S. aureus*), streptococci, and enterobacteria are the most frequently found causative agents of EBE [9, 10]. Infection of indwelling catheters or prosthetic devices with *S. aureus* is associated with bacterial endocarditis and is the major cause of EBE in Europe and the United States of America. Diabetes mellitus and immunosuppression are additional risk factors for a systemic bacterial infection and thus EBE [9, 10].

Pathogens causing EBE have to pass the blood–ocular barrier, namely, the blood–aqueous barrier and the blood–retinal barrier. The blood–aqueous barrier consists of endothelial cells of the retina capillary vessels and is in direct contact with pericapillary glial cells. The outer blood–retinal barrier consists of the RPE cells and the underlying Bruch’s membrane and separates the neuronal retina from the fenestrated choriocapillaris. The barrier function of RPE cells is accomplished by tight junctions. The flow of nutrients and macromolecules from the blood to the retina is thereby regulated and the entry of pathogens and immune cells to the eye is restricted [11]. In addition, RPE cells are immune competent. They produce several cytokines including interleukin-1 (IL-1) and tumor necrosis factor alpha (TNFα), can activate T cells and are able to restrict the growth of pathogens [12]. The antimicrobial capacity of the RPE has been analyzed by several groups. For example, RPE cells are susceptible to different viruses such as Herpes simplex virus, cytomegalovirus, adenovirus types 1 and 7, and measles virus [13]. Furthermore, IFN-γ activated RPE cells can restrict the growth of *T. gondii* and CMV, which are both capable to induce retinitis. In general, indoleamine 2,3-dioxygenase-1 (IDO1) and inducible nitric oxide synthase (iNOS) were identified as prominent IFN-γ induced effector mechanisms against CMV [7].

iNOS can be expressed in retinal pigment epithelial cells from mice and rats [14, 15]. The observation that iNOS-deficient mice have an increased CMV load in the retina after CMV infection is a hint for a functional involvement of iNOS in the microbial defense [16]. Human RPE (hRPE) cells express iNOS; however, iNOS induction by cytokines or by environmental factors differs between species [17, 18]. In addition to its role in antimicrobial response, iNOS has also been found to be involved in the development of ischemic retinopathies [19], age-related macular degeneration [20], chronic glaucoma [21], and diabetic retinopathy [22]. In contrast to the antiviral effects mediated by iNOS in murine cells, Bodagi et al. found that iNOS is not important in the defense of hRPE cells against CMV [7]. Instead, IFN-γ induced IDO1 served as an antiviral defense mechanism in native hRPE cells. In accordance Nagineni et al. described, the IFN-γ stimulation of primary hRPE cells induced IDO1, which inhibited the growth of *T. gondii* [6].

Thus, IDO1- and iNOS-mediated effects against *T. gondii* and CMV have been studied intensively. However, relatively few information is available regarding the RPE cell-mediated antimicrobial defense against bacteria, especially with the focus on *S. aureus* causing EBE. Healthy and diabetic C57BL/6 mice develop EBE after intravenous infection with *S. aureus* [23]. An *in vitro* infection with *S. aureus* results in disruption of the tight junctions of hRPE cells [10]. Furthermore, hRPE cells can phagocytose *S. aureus* and present staphylococcal superantigens to T cells [24, 25]. The defense mechanisms against *S. aureus* mediated by the RPE are unclear; however, respiratory burst is likely not involved [24]. Therefore, we aimed to analyze the role of iNOS and IDO1 in the defense against *S. aureus* in hRPE cells.

## Materials and methods

### Cells, media, and reagents

Human retinal pigment epithelial (hRPE) cells (ARPE-19, ATCC, Wesel, Germany), as well as human foreskin fibroblasts (HFF) (ATCC, Wesel, Germany) and human glioblastoma cells (86HG39) were cultured in Iscove’s modified Dulbecco’s medium (IMDM) (Gibco, Grand Island, USA), supplemented with 5% heat-inactivated fetal calf serum (FCS). Cells were cultured in culture flasks (Costar, Cambridge, USA) in a humidified incubator (37 °C, 5% CO_2_). Cells were passaged weekly in 1:10 ratios using trypsin/EDTA (Gibco, Grand Island, USA). Hypoxia growth experiments were carried out using a HERAcell 150 I CO_2_ incubator (Thermo Fisher Scientific, Carlsbad, USA) or the Anoxomat™ system (Mart Microbiology B. V., Drachten, Netherlands) with 1–10% O_2_ and 5% CO_2_. Mycoplasma contamination was regularly excluded by culture methods and PCR.

A tryptophan-auxotrophic *Staphylococcus aureus* isolate was used. This *S. aureus* isolate was obtained from a routine diagnostic specimen [26]. *S. aureus* was grown on brain heart infusion agar containing 5% sheep blood (Difco, Hamburg, Germany) at 37 °C in 5% CO_2_-enriched atmosphere overnight.

*Toxoplasma gondii* tachyzoites (ME49 strain, ATCC, Wesel, Germany) were maintained in HFF in IMDM containing 5% FCS. Extracellular tachyzoites were harvested from culture supernatants by centrifugation, resuspended in tryptophan free RPMI, counted and used as indicated for infection experiments.

### Stimulation of hRPE cells

For infection experiments as well as for the determination of IDO1 and iNOS activity, hRPE cells were seeded in 96-well plates (3 × 10^4^ cells per well) or 24-well plates (3 × 10^4^, 1 × 10^5^, 3 × 10^5^ or 5 × 10^5^ cells per well) and stimulated with recombinant human IFN-γ (0–2000 U/ml) (R&D Systems, Minneapolis, USA) and/or recombinant human IL-1β (100 U/ml) (R&D Systems, Minneapolis, USA) and/or recombinant human TNFα (100 U/ml) (R&D systems, Minneapolis, USA) for 72 h in a humidified incubator (37 °C, 5% CO_2_). In the iNOS activity kinetic experiments, supernatants were harvested after 0, 24, 48, 72, or 96 h and analyzed with the nitric oxide assay. Subsequent infection experiments as well as IDO1 and iNOS activity measurements were performed with pre-stimulated cells.

In some experimental groups, the NOS inhibitor *N*^G^-monomethyl-l-arginine (N^G^MMA) (Merck, Darmstadt, Germany) (100 µg/ml) or the competitive IDO inhibitor 1-l-methyl-tryptophan (1-MT) (Sigma-Aldrich, St. Louis, USA) (1.5 mM) were used.

### Kynurenine assay

The enzymatic activity of IDO1 directly correlates with the concentration of kynurenine in supernatants of tissue culture cells, and therefore, the measurement of kynurenine can be used to determine IDO1 activity [27]. The kynurenine content of supernatants from unstimulated or stimulated cells was analyzed using 4-(dimethylamino) benzaldehyde (Ehrlich´s reagent) as described before [27].

### Nitric oxide assay

Nitrite accumulation in the supernatant of cultured cells was used as an indicator of nitric oxide production and was determined by the Griess reaction [28]. In brief, 100 μl cell-culture supernatant of pre-stimulated hRPE cells (for 72 h) was mixed with 100 μl Griess Reagent (0.1% *N*-(1-naphthyl)ethylenediamine in purified water and 2.5% sulfonamide in 15% hydrochloric acid in a 1:1 ratio; Merck, Darmstadt, Germany). After an incubation time of 15 min, the absorbance was measured at 540 nm (TECAN Sunrise microplate reader, Crailsheim, Germany). The amount of nitrite accumulation was determined using a calibration curve of graded concentrations of sodium nitrite.

### Determination of bacterial growth

A 24-h-old *S. aureus* colony was picked, resuspended in PBS (Gibco, Grand Island, USA), and serial diluted. After pre-stimulation with cytokines for 72 h, hRPE cells (cultured in 96 flat-bottom culture plates) were infected with 10 µl of the bacterial dilution containing 10–100 colony forming units (cfu). Alternatively, conditioned medium from stimulated hRPE cultures (harvested 72 h after cytokine stimulation) was inoculated with the same amount of bacteria. Infected cultures were incubated in a humidified incubator (37 °C, 5% CO_2_) for 16 h. Bacterial growth was monitored by measuring the optical density of resuspended cultures at 620 nm (TECAN Sunrise microplate reader, Crailsheim, Germany) [26].

### Determination of parasite growth

After pre-stimulation for 72 h, hRPE cells were infected with 2 × 10^4^*T. gondii* tachyzoites per well. *T. gondii* growth was determined by the ^3^H-uracil incorporation method as described before [26, 29]. In brief, ^3^H-uracil (0.33 μCi per well) was added 48 h postinfection. Cultivation was stopped after additional 24 h by freezing. Parasite growth was determined by measuring incorporated ^3^H-uracil using liquid scintillation spectrometry (1205 Betaplate, PerkinElmer, Jugesheim, Germany).

### Real-time PCR

hRPE cells were seeded into 6-well plates (10^6^ hRPE cells per well) and stimulated with combinations of the respective cytokines IFN-γ (500 U/ml), IL1-β (100 U/ml), TNFα (100 U/ml), and N^G^MMA (100 µg/ml) or left untreated for 24 h in a humidified incubator at 37 °C and 5% CO_2_ [8]. For sample collection, the medium was aspirated and cells were detached in PBS by scraping. Total RNA was extracted according to the TRI Reagent protocol (Merck, Darmstadt, Germany). RNA was dissolved in UltraPure™ distilled water (Thermo Fisher Scientific, Carlsbad, USA) and RNA concentration was determined via NanoDrop (Thermo Fisher Scientific, Carlsbad, USA). Reverse transcription of 2 µg total RNA to cDNA was performed with M-MLV reverse transcriptase and oligo(dT)_12–18_ primers (Thermo Fisher Scientific, Carlsbad, USA). PCR primers to amplify the genes of interest are listed in Table 1. Real-time PCR was performed with the Takyon NoRox Probe MasterMix dTTP (Eurogentec, Lüttich, Belgium) on a Bio-Rad CFX96 Touch Real-Time PCR Detection system (Bio-Rad Laboratories, Hercules, USA). All reactions, including non-template contamination controls, were performed in duplicates. Each well of a multiplate 96-well PCR plate contained 5 μl cDNA template, 12.5  μl Takyon NoRox Probe Master Mix dTTP, 0.3 μl primer (20 μM each), 0.5 µl probe (10 µM), and 6.4 μl H_2_O for a total reaction volume of 25 μl (Table 1). The PCR conditions were 7 min at 95 °C and 40 cycles of each 94 °C for 20 s and 60 °C for 1 min.

**Table 1:** Real-time PCR primers for the detection of human beta-actin, nitric oxide synthase, and indoleamine 2,3-dioxygenase-1 transcripts

Gene of interest	Gene ID	Probe	Sequence [5′ → 3′]		Length
β-Actin	ENSG00000075624	#64	fw	ccaaccgcgagaagatga	18
			rv	ccagaggcgtacagggatag	20
iNOS (inducible, NOS2)	ENSG00000007171	#16	fw	tcttcctggtttgactgtcctta	23
			rv	gctcagatgttcttcactgtgg	22
eNOS (endothelial, NOS3)	ENSG00000164867	#67	fw	gactgaaggctggcatctg	19
			rv	ccatgttactgtgcgtccac	20
nNOS (neuronal, NOS1)	ENSG00000089250	#39	fw	tgggagactgaggtggttct	20
			rv	gtactcagtgcatcccgtttc	21
IDO1	ENSG00000131203	#9	fw	cgccttgcacgtctagttct	20
			rv	ttggcagtaaggaacagcaat	21
IDO2	ENSG00000188676	#4	fw	ttcctcaccatgggttatgtc	21
			rv	gaagggcaagattccttgg	19

### Western blot analysis

6,75 x 10^6^ hRPE cells were incubated in cell-culture flasks (Costar, Cambridge, USA) in the absence or presence of IFN-γ (500 U/ml), IL-1β (100 U/ml), and TNFα (100 U/ml) for 24 h. The supernatant was discarded and the cell monolayer was washed three times with cold PBS. The cells were detached by scraping in 200 µl PBS containing a protease inhibitor cocktail (Roche Diagnostics, Mannheim, Germany). Thereafter, the cells were lysed by three freeze/thaw cycles, centrifuged and the cell extract was stored at − 80 °C. IFN-γ (500 U/ml) stimulated human glioblastoma cell extracts served as a control. The protein amount was determined via Bradford assay (Bio-Rad Laboratories, Hercules, USA). Electrophoretic separation of proteins (30 µg protein per lane) was done with 10% NuPAGE Novex Bis–Tris Mini gels in the appropriate electrophoresis system (Thermo Fisher Scientific, Carlsbad, USA). Separated proteins were semi-dry blotted on nitrocellulose membranes (CarboGlas, Schleicher & Schüll, Dassel, Germany). After blocking the membranes in 5% (w/v) skim milk powder in PBS for 1 h at room temperature, the nitrocellulose membranes were incubated for 1.5 h at room temperature in the respective primary antibodies diluted in 0.5% (w/v) skim milk powder in TBS: anti-β-actin antibody (1:5000, Sigma, St. Louis, USA) or anti-human-IDO1 antibody (1:500, Merck, Darmstadt, Germany). Thereafter, the membranes were washed with PBS for three times (5 min) and incubated for 2 h at room temperature with goat anti-mouse HRP-conjugated (IDO1) or goat anti-rabbit HRP-conjugated (ß-actin) IgG (1:10,000-70,000, Jackson Immuno Research Laboratories, Dianova, Hamburg, Germany), diluted in 0.5% (w/v) skim milk powder in PBS. After additional washes with PBS, bands were detected by enhanced chemiluminescence (Amersham Pharmacia Biotech, Freiburg, Germany).

### Statistical analysis

All experiments were performed in duplicates or triplicates (as indicated in the figure legends) and data are given as mean ± SEM of a minimum of three independent experiments. For statistical analysis the unpaired two-tailed students *t* test was used and significant differences were marked with asterisks (*p < 0.05; **p < 0.001; ***p < 0.0001). The statistical analysis was performed with GraphPad Prism software.

## Results

### Indoleamine 2,3-dioxygenase-1 mediates antimicrobial functions in hRPE cells

**Fig. 1:**
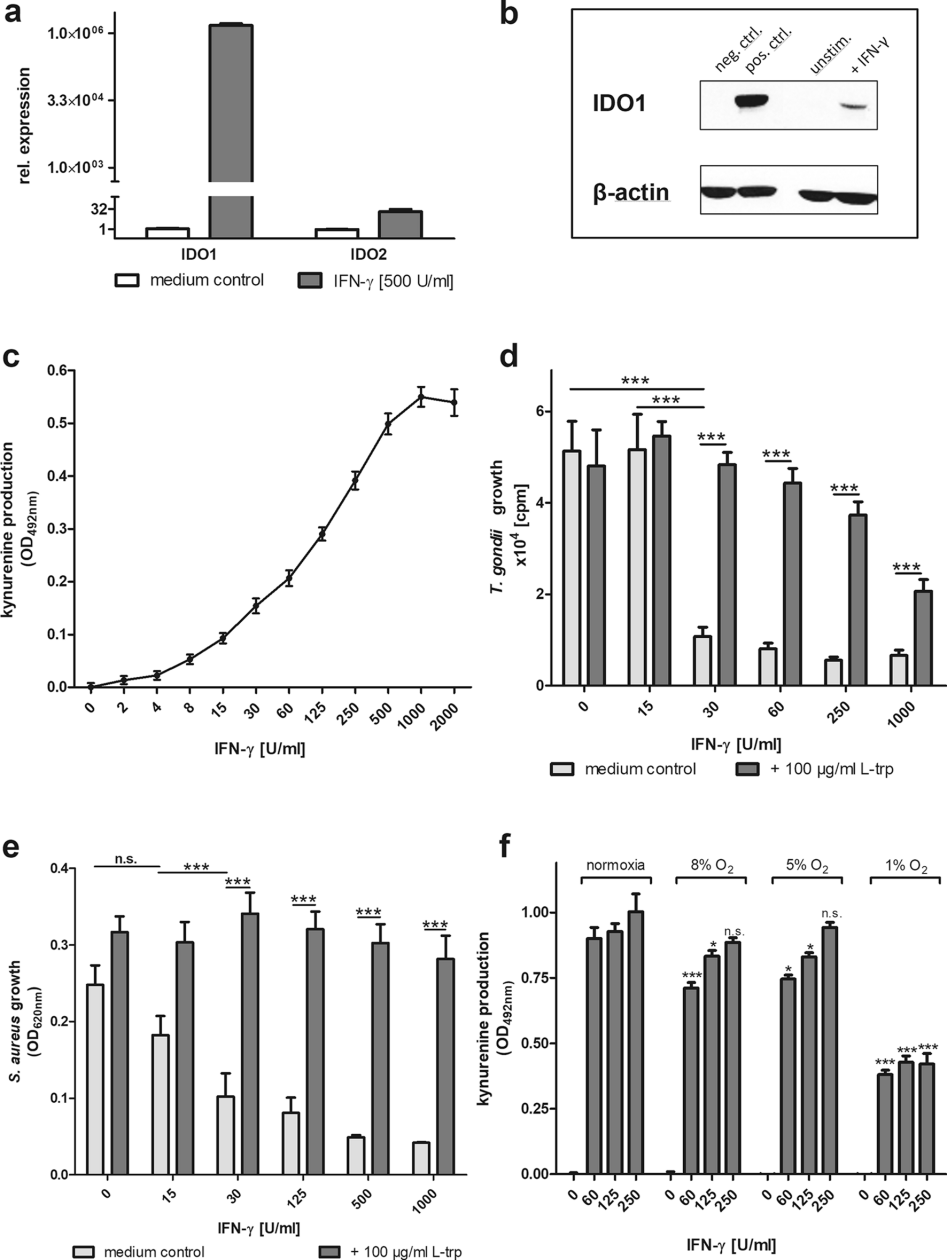
Activity of indoleamine 2,3-dioxygenase (IDO) in human retinal pigment epithelial (hRPE) cells. **a** Relative expression of IDO1 and IDO2 in unstimulated (medium control) or IFN-γ (500 U/ml for 24 h) stimulated hRPE cells, detected by real-time PCR. **b** Exemplary western blot analysis showing IDO1 negative and positive control (human glioblastoma lysates) and IDO1 and β-actin protein in unstimulated and IFN-γ (500 U/ml for 24 h) stimulated hRPE cells. **c** 3 × 10^4^ hRPE cells were stimulated in 96-well plates with indicated concentrations of human IFN-γ (0–2000 U/ml) in the presence of l-tryptophan (L-trp; 100 µg/ml). After 72 h, the cell-culture supernatants were harvested and the kynurenine content was determined by the use of Ehrlich’s reagent. (d + e) 3 × 10^4^ hRPE cells pre-stimulated with IFN-γ for 72 h. **d** Pre-stimulated hRPE cell cultures were infected with *Toxoplasma gondii* (1 × 10^5^ ME49 tachyzoites/well) or (**e**) *Staphylococcus aureus* (10–100 cfu/well) without additional l-tryptophan (medium control) or with l-tryptophan (100 µg/ml) supplementation. Parasite growth was determined via the ^3^H-Uracil method and the bacterial growth was detected by the optical density at 620 nm (OD_620 nm_). **f** 3 × 10^4^ hRPE cells were stimulated with indicated IFN-γ concentrations under atmospheric oxygen concentration (normoxic) or hypoxic (8% O_2_, 5% O_2,_ or 1% O_2_) conditions in the presence of l-tryptophan (100 µg/ml). Ehrlich’s reagent was used to measure the kynurenine content. Data are given as mean ± SEM of two (**a**), one (**b**), three (**c**), five (**d**), four (**e**), or three (**f**) experiments, each performed in triplicate. Significant differences to the unstimulated or normoxic group are indicated with asterisks (*n.s.* not significant, *p ≤ 0.05, **p ≤ 0.001, ***p ≤ 0.0001). The unpaired, two-tailed student’s *t* test was used

Our aim was to analyze antibacterial effectors in IDO-positive human retinal pigment epithelial (hRPE) cells, since IDO-mediated antibacterial effects have been described in human cells before [6, 7]. Hence, we stimulated hRPE cells with IFN-γ and found a strong expression of IDO1 mRNA (10^6^ fold higher than in unstimulated cells), whereas IDO2 mRNA was only marginally induced (32-fold) and thus might be neglectable (Fig. 1a, b).

IDO1 activity was assured by the measurement of kynurenine, the product of IDO1-mediated tryptophan degradation. For this purpose, hRPE cells were stimulated in a tryptophan-enriched cell-culture medium with different amounts of IFN-γ for 72 h and the accumulated amount of kynurenine in the cell-culture supernatants was quantified by the use of Ehrlich´s reagent. IDO1 activity was clearly dependent on the IFN-γ dose used (Fig. 1c) and even 4 U/ml IFN-γ were sufficient to induce a significant increase (*p* > 0.001) of kynurenine production. To further prove that IDO1 is the responsible antimicrobial effector molecule in stimulated hRPE cells, we performed additional experiments using the IDO1-specific inhibitor 1-methyl-l-tryptophan (1-MT). These data are included in the electronic supplementary material (ESM 1). 1-MT treatment of IFN-γ stimulated hRPE cells inhibits IDO1 activity, as detected by kynurenine measurement (ESM 1a).

Next, we tested whether IDO1 could sufficiently degrade the conventional tryptophan amounts in the IMDM cell-culture medium to inhibit the growth of pathogens. It has been shown before that IDO1 inhibits RH (type I) strain *Toxoplasma gondii* in hRPE cells [6]. Herein, we tested the efficiency of IDO1 activity against ME49 parasites, since type II strains are the most frequently found *T. gondii* strains in patients [30]. IFN-γ pre-stimulated hRPE cells were infected with *T. gondii* and the intracellular growth was quantified after 3 days using the ^3^H-uracil method. *T. gondii* tachyzoites grew in unstimulated hRPE cells, but the presence of 30 U/ml IFN-γ was sufficient to inhibit the parasite growth significantly (Fig. 1d). This antiparasitic effect was due to IDO1-mediated tryptophan degradation, since the supplementation of l-tryptophan at the timepoint of infection abrogated the effect (Fig. 1d). Interestingly, there was no full recovery of *T. gondii* growth in hRPE cells that were pre-treated with high doses of IFN-γ.

Since stimulated hRPE cells displayed an effective IDO1 activity, we then checked their ability to inhibit *S. aureus* growth as well. We used a patient-derived tryptophan auxotroph *S. aureus* strain to infect hRPE cells that were pre-stimulated with different amounts of IFN-γ for 72 h. The bacterial growth was analyzed 16 h postinfection by measuring the optical density. Bacteria grew in the presence of unstimulated cells. *S. aureus* growth was significantly inhibited by hRPE cells pre-stimulated with 30 U/ml IFN-γ or more (*p* < 0.0001, Fig. 1e). The antibacterial effect could be ascribed to IDO1-mediated tryptophan degradation, since the supplementation of l-tryptophan at the timepoint of infection allowed an unrestricted bacterial growth (Fig. 1e). Furthermore, the IDO-specific inhibitor 1-MT abrogated the antibacterial effect as well (ESM 1b).

hRPE cells are localized in a region of the eye with a physiologic oxygen concentration of approx. 8% O_2_ [35]. Since IDO1 activity is dependent on the presence of oxygen, we analyzed the kynurenine production in hRPE cells under different oxygen conditions. hRPE cells were incubated under normoxia, 8% O_2_, 5% O_2,_ or 1% O_2_ in the presence of different IFN-γ concentrations. IDO1 activity was again measured on the basis of the kynurenine content in the cell-culture supernatants. At 8% O_2,_ IDO1 activity in hRPE cells is slightly reduced at lower IFN-γ concentrations (60 and 125 U/ml). However, there was no significant impact on IDO1 activity when the hRPE cells were treated with higher amounts of IFN-γ (250 U/ml) (Fig. 1f). In contrast to this, the hypoxic environment of only 1% O_2_ resulted in a significant decrease of IDO1 activity, even in the presence of higher IFN-γ concentrations (Fig. 1f). In addition, the presence of 1% O_2_ results in a strong inhibition of IFN-γ induced antibacterial effect, while in the presence of 8% O_2,_ a more or less complete antibacterial effect of IFN-γ was detected (ESM 1c).

### Inducible NO synthase is induced in hRPE cells by cytokine co-stimulation

**Fig. 2:**
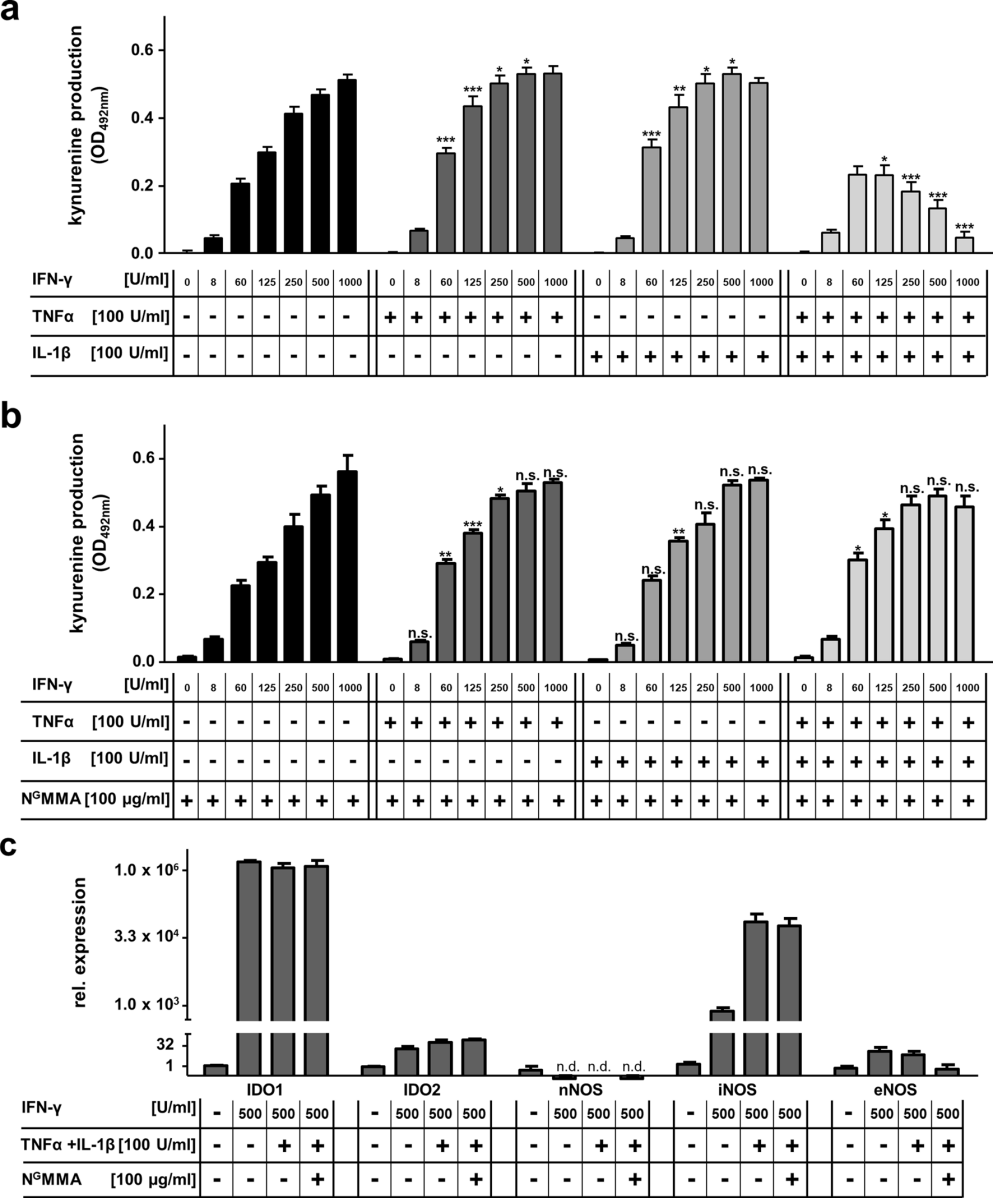
Influence of the inducible NO synthase (iNOS) on indoleamine 2,3-dioxygenase-1 (IDO1) activity in human retinal pigment epithelial (hRPE) cells. **a** 3 × 10^4^ hRPE cells were stimulated in 96-well plates with indicated concentrations of human IFN-γ (0–1000 U/ml) in the presence of 100 µg/ml l-tryptophan and in the absence (−) or presence (+) of IL-1β (100 U/ml) and TNFα (100 U/ml). After 72 h, the cell-culture supernatants were harvested and the kynurenine content was determined by the use of Ehrlich´s reagent. **b** Same experimental setting with additional supplementation of the iNOS inhibitor N^G^MMA (100 µg/ml). **c** Relative expression of IDO1, IDO2, nNOS, iNOS, and eNOS detected by real-time PCR 24 h after stimulation. Data are given as mean ± SEM of five (**a**, **b**) or two (**c**) experiments, each performed in triplicate. Significant differences to the unstimulated group were marked with asterisks (*n.s.* not significant, *n.d.* not detected, *p ≤ 0.05, **p ≤ 0.001, ***p ≤ 0.0001). The unpaired, two-tailed student’s *t* test was used

The antiparasitic effect against RH strain *T. gondii* tachyzoites is not only provoked by IFN-γ, but also by other proinflammatory cytokines and synergistic effects have been described [26]. Therefore, we co-stimulated 3 × 10^4^ hRPE cells with IFN-γ and IL-1β or TNFα, respectively, in 96-well plates. This co-stimulation leads to a slight increase of IDO1 activity, as observed by the formation of kynurenine in the cell-culture supernatants obtained after stimulation with 60–500 U/ml IFN-γ (Fig. 2a). Therefore, we expected a synergistic effect of all three cytokines. However, unexpectedly, the co-stimulation of IFN-γ, IL-1β, and TNFα resulted in a significant reduction of the kynurenine production at IFN-γ concentrations above 125 U/ml. Such an inhibition of IDO1 activity could be the result of the induction of iNOS. To find out whether iNOS is involved in the inhibition of IDO1 activity, we repeated the experiment in the presence of N^G^-monomethyl-l-arginine (N^G^MMA), a competitive inhibitor that reduces the iNOS-mediated conversion of arginine to nitric oxide (NO). N^G^MMA had no effect on IFN-γ-induced IDO1 activity (Fig. 2b). In addition, the presence of N^G^MMA did not influence IDO1, which was co-stimulated by IFN-γ and IL-1β or IFN-γ and TNFα (Fig. 2b). Interestingly, N^G^MMA supplementation restored IDO1 activity after co-stimulation with all three cytokines (Fig. 2b). Therefore, it seems probable that the co-stimulation of hRPE cells with IFN-γ, IL-1β, and TNFα indeed induced iNOS in hRPE cells, which was inhibited by N^G^MMA. Furthermore, NO, the product of iNOS activity must have been the causing agent for the IDO1 inhibition.

Nevertheless, we wanted to confirm that indeed, iNOS and not the other NO-synthases (endothelial or neuronal NOS, eNOS, and nNOS, respectively) were responsible for the formation of NO. Real-time PCR studies revealed a highly significant increase of iNOS in hRPE cells after co-stimulation with IFN-γ, IL-1β, and TNFα (Fig. 2c), which was not affected by the addition of the competitive inhibitor N^G^MMA as expected (Fig. 2c). Due to the low mRNA expression of the other NO-synthases, we assume that nNOS and eNOS do not play a major role in the inhibition of IDO1 in hRPE cells (Fig. 2c). In sum, the co-stimulation of IFN-γ, IL-1β, and TNFα results in the induction of NO producing iNOS, which inhibits the IDO1-mediated degradation of tryptophan to kynurenine.

**Fig. 3:**
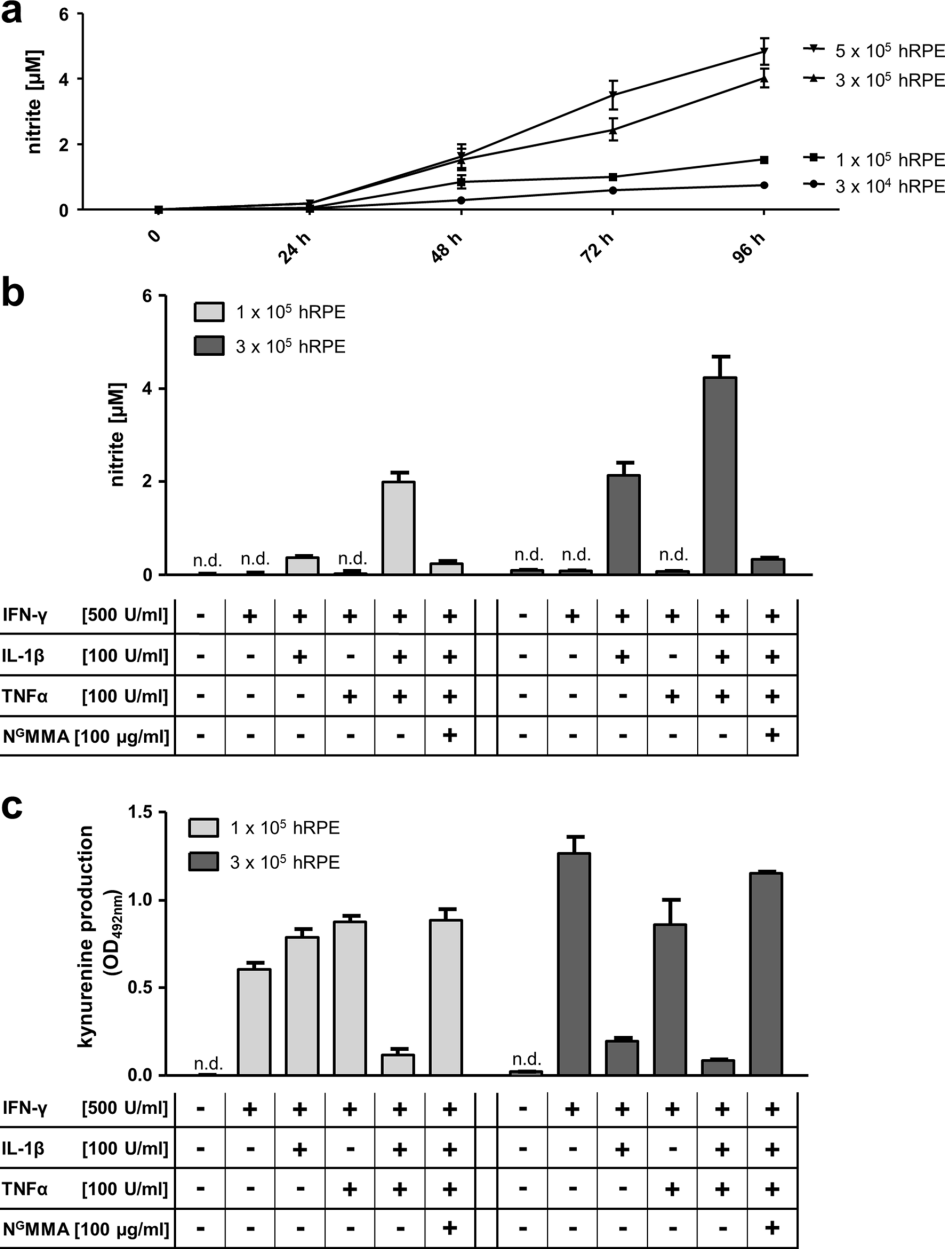
Optimized NO production and impact of high NO levels on IDO1. **a** 3 × 10^4^, 1 × 10^5^, 3 × 10^5^, or 5 × 10^5^ hRPE cells were stimulated in 24-well plates with human IFN-γ (500 U/ml), IL-1β (100 U/ml), and TNFα (100 U/ml) for 0–96 h. From each probe, 100 µl cell-culture supernatant was added to 100 µl Griess Reagent (0.1% *N*-(1-napthyl)ethylendiamin [in aqua dest. plus 2.5% sulfanilamid (in 15% HCl) in equal parts] and the nitrite was quantified by measuring the optical density at 540 nm. **b** 1 × 10^5^ or 3 × 10^5^ hRPE cells were stimulated in the presence (+) or absence (−) of IFN-γ (500 U/mL), IL-1β (100 U/ml), TNFα (100 U/ml), or N^G^MMA (100 µg/ml). After 72 h, 100 µl cell-culture supernatant was added to 100 µl Griess Reagent (0.1% *N*-(1-Napthyl)ethylendiamin [in aqua dest. plus 2.5% Sulfanilamid (in 15% HCl) in equal parts] and the nitrite was quantified by measuring the optical density at 540 nm. **c** 1 × 10^5^ or 3 × 10^5^ hRPE cells were stimulated in the presence (+) or absence (−) of IFN-γ (500 U/mL), IL-1β (100 U/ml), TNFα (100 U/ml), or N^G^MMA (100 µg/ml). After 72 h, the cell-culture supernatants were harvested and the kynurenine content was determined by the use of Ehrlich’s reagent. Data are given as mean ± SEM of two experiments, each performed in triplicate

Next, we verified nitric oxide production indirect by detection of nitrite in the supernatant of IFN-γ, IL-1β, and TNFα co-stimulated hRPE cells by the use of Griess Reagent. We could not detect significant amounts of NO in 3 × 10^4^ co-stimulated hRPE cells in 96-well plates (data not shown). However, higher levels of NO accumulated in cell-culture supernatants of 1 × 10^5^ up to 5 × 10^5^ co-stimulated hRPE cells in 24-well plates over time. Therefore, the amount of NO is dependent on the cell number used in the experiments (Fig. 3a).

We continued with the 24-well-plate experimental setting and analyzed the NO production in 1 × 10^5^ differently stimulated hRPE cells. IFN-γ stimulation did not exceed the NO production of the negative control, whereas a combinatorial treatment of hRPE cells with IFN-y and IL-1β leads to iNOS activity (Fig. 3b). In contrast, no NO was detectable after the combinatorial treatment with IFN-y and TNF-α (Fig. 3b). Interestingly, as expected, the stimulation with all three cytokines resulted in an intense formation of NO and the addition of N^G^MMA prevented the generation of NO (Fig. 3b). All observations were enhanced by the usage of 1 × 10^5^ hRPE cells (Fig. 3b).

Since not only the co-stimulation with all three cytokines resulted in a significant NO production in the modified experimental system, but also the combination of IFN-γ and IL-1β alone, we were interested in whether the latter stimulation had an influence on IDO activity. Although IDO activity has not been influenced by IFN-γ and IL-1β stimulation in 3 × 10^4^ hRPE cells (Fig. 2a) and in 1 × 10^5^ hRPE cells (Fig. 3c), IDO activity was clearly inhibited by IFN-γ and IL-1β-induced NO production in 3 × 10^5^ stimulated hRPE cells (Fig. 3c). These results indicate that a sufficient NO concentration is indispensable for a significant inhibition of IDO activity.

### Influence of iNOS activity on indoleamine 2,3-dioxygenase-1 in human retinal pigment epithelial cells

**Fig. 4:**
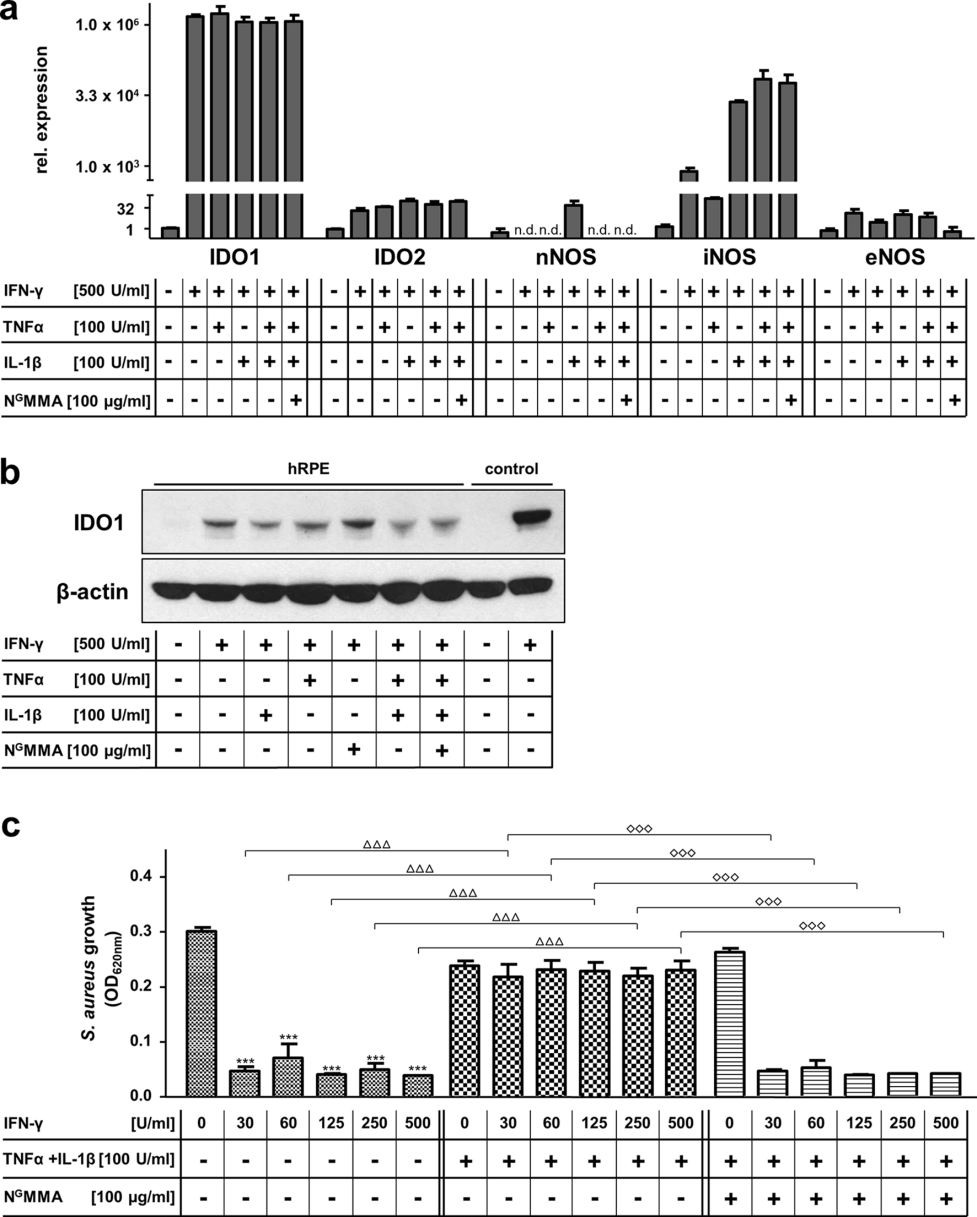
Influence of iNOS activity on indoleamine 2,3-dioxygenase-1 in human retinal pigment epithelial cells. **a** Relative expression of IDO1, IDO2, neuronal (nNOS), inducible (iNOS), and endothelial nitric oxide synthase (eNOS) 24 h poststimulation as determined by real-time PCR. hRPE cells were untreated or stimulated with combinations of IFN-γ (500 U/ml), IL-1β (100 U/ml), TNFα (100 U/ml), and N^G^MMA (100 µg/ml) as indicated. **b** Western blot analysis of IDO1 and β-actin protein expression by hRPE cells in the presence (+) or absence (−) of IFN-γ (500 U/ml), IL-1β (100 U/ml), or TNFα (100 U/ml). Unstimulated and IFN-γ (500 U/ml) stimulated human glioblastoma cell lysates were used as controls, respectively. **c** 3 × 10^4^ hRPE cells pre-stimulated with IFN-γ and/or IL-1β and TNFα (100 U/ml each) for 72 h were infected with *Staphylococcus aureus* (10–100 cfu/well). The bacterial growth was detected by measurement of the optical density at 620 nm after 16 h. Data are given as mean ± SEM of two (**a**), one (**b**) or three (**c**) experiments, each performed in triplicate. Significant inhibition of bacterial growth in IFN-γ stimulated hRPE cells is marked with asterisks (*p ≤ 0.05, **p ≤ 0.001, and ***p ≤ 0.0001). Significant inhibition of the antibacterial effect by co-stimulation with IL-1β and TNFα is marked with triangles. Significant recovery of the antibacterial effector mechanism via N^G^MMA supplementation is marked with diamonds. The unpaired, two-tailed student’s *t* test was used

Next, we elucidated whether iNOS activity inhibits IDO1 on transcriptional, translational, or post-translational level. In real-time analyses, IDO1 mRNA induction was detected upon IFN-γ stimulation (Fig. 4a). Neither combinatorial stimulation with IFN-γ/IL-1β, nor IFN-γ/TNFα, nor IFN-γ/IL-1β/TNFα lead to a reduced IDO1 mRNA expression (Fig. 4a).

Western blot analyses revealed no reduction of IDO1 protein levels upon IFN-γ/IL-1β, IFN-γ/TNFα or IFN-γ/IL-1β/TNFα stimulation (Fig. 4b). As expected, N^G^MMA had no effect on IDO1 protein levels as well.

Finally, we wanted to elucidate whether iNOS-generated NO is sufficient to have a potential role during EBE. Since NO inhibits the IDO1-mediated kynurenine production, we wanted to find out whether IDO1-mediated antibacterial effects are also abolished. Therefore, IFN-γ or IFN-γ/IL-1β/TNFα pre-stimulated hRPE cells were infected with *S. aureus* and the bacterial growth was determined after additional 16 h by measurement of the optical density. Bacterial growth was inhibited in IFN-γ pre-stimulated cells as before (Fig. 3c). Interestingly, the co-stimulation with IFN-γ, IL-1β, and TNFα impeded the IDO1 antibacterial activity, which allowed an unhindered *S. aureus* growth (Fig. 4c). The presence of N^G^MMA during the stimulation period sufficiently neutralized this effect (Fig. 4c).

## Discussion

The eye is an immune-privileged site. This privilege is established by the RPE. The RPE cells play a crucial role in retinal physiology, in the induction of immunosuppression and in antimicrobial defense [4]. Eye infections cause harm to vision. The work of several research groups focusses especially on CMV and *T. gondii*-caused infections, since both pathogens induce numerous eye infections.

For example, Noguereia et al. described barrier dysfunction of human RPE cells due to altered tight junctions after in vitro infection with *T. gondii* RH strain tachyzoites [31]. Delaire et al. found out that IFN-γ activated native Lewis rat RPE cells are able to inhibit the growth of *T. gondii* (RH strain) tachyzoites [5]. Furthermore, stimulation with TNFα alone inhibited *T. gondii* growth slightly. A combined treatment of native Lewis rat RPE cells with IFN-γ and TNFα resulted in a synergistic effect. Further data from this group suggest that iNOS was not involved in the toxoplasmostatic effect [31]. In accordance with data from Delaire et al., Nagineni et al. found that primary human RPE lines stimulated with IFN-α, IFN-β, IFN-γ, and TNFα are able to inhibit *T. gondii* growth (RH strain), while IFN-γ was the most efficient cytokine [5, 6]. Interestingly, both groups hold the induction of the tryptophan-degrading enzyme IDO1 responsible for the antiparasitic effect observed in IFN-γ stimulated human RPE cells [5, 6]. In detail, they detected IDO1 mRNA and protein in IFN-γ-stimulated RPE cells. Tryptophan supplementation dramatically reduced the antiparasitic effect of IFN-γ-stimulated RPE cells against *T. gondii*. Type I *T. gondii*, such as the RH strain, are highly virulent in mice. However, the majority of *T. gondii* isolates are type II strains, whereas type I strains are rarely isolated in humans [30]. Chronically infected patients, especially those with toxoplasmic chorioretinitis, are most frequently affected by type II strains [30]. Due to this reason, we used the *T. gondii* type II strain ME49 in our experimental setup. We confirmed the production of IDO1 mRNA and protein in IFN-γ stimulated hRPE ARPE19 cells. Since these cells express IDO1 as well as iNOS simultaneously after IFN-γ, IL-1β, and TNFα stimulation, we conclude that in this respect, the ARPE19 cell line used in this study is comparable to native hRPE cells [6, 7]. Beyond that, we could show that there was no noteworthy amount of IDO2 mRNA in IFN-γ-stimulated hRPE cells. In addition, we were able to prove IDO1 enzyme activity, since we measured an accumulation of kynurenine in the cell-culture supernatant of IFN-γ-stimulated hRPE cells. Furthermore, we could ascribe the IFN-γ-induced antiparasitic effect to IDO1, since we found that tryptophan supplementation at the timepoint of infection results in a nearly complete recovery of *T.* *gondii* ME49 tachyzoite growth. However, we observed that there was no full recovery of *T. gondii* growth by supplemental tryptophan in hRPE cells pre-treated with high doses of IFN-γ. This might be a hint for the presence of toxic amounts of kynurenine or kynurenine-derived metabolites that affect the parasites, but not *S. aureus* or host cells [32].

*Staphylococcus aureus* is the most frequent causative agent in patients with endogenous endophthalmitis (EBE). We revealed that IDO1 activity inhibits *S. aureus* growth in human brain microvascular endothelial cells [33]. Here, we found that IFN-γ-activated hRPE cells restrict the growth of *S. aureus* and that tryptophan supplementation abrogates this antibacterial effect. These data indicate that hRPE cells are antimicrobial effector cells that might influence EBE. The cleavage of tryptophan by IDO1 is critically dependent on the availability of oxygen [34]. Under physiologic conditions, the RPE has an oxygen saturation of approximately 8% O_2_ [35]. Therefore, we incubated hRPE cells under normoxic (20% O_2_), physiologic (8% O_2_) and hypoxic (1% O_2_) conditions. We found that IDO1 activity was strongly reduced during cultivation under the hypoxic oxygen concentration (1% O_2_), while IDO1 activity was only marginally influenced in hRPE cells cultivated under physiologic oxygen levels (8% O_2_) in comparison with normoxia (20% O_2_). This means that IDO1 is active at physiologic oxygen concentration in the hRPE cell monolayer in vivo, but might be affected under pathophysiological conditions during inflammation.

In previous experiments with human glioblastoma cells and alveolar epithelial cells, we found that TNFα and IL-1β, respectively, did not induce IDO1 directly, but required IFN-γ to synergistically induce IDO1 [26, 36]. Co-stimulation of hRPE cells with IFN-γ and TNFα or IL-1β increased IDO1 activity significantly. However, a combined stimulation with IFN-γ, IL-1β, and TNFα resulted in a strong inhibition of IFN-γ-induced IDO1 activity, especially at higher IFN-γ doses. Since nitric oxide is capable of inhibiting IDO1 activity [37], we assumed that iNOS induction in hRPE cells by this cytokine cocktail might be responsible for the observed IDO1 inhibition.

Earlier publications described iNOS transcription, expression, and activity in human native RPE cells as well as in the RPE cell line ARPE19 [7, 17, 22]. In this context, iNOS activity was induced by stimulation with different cytokines (for example, with IFN-γ, TNFα, and/or IL-1β). In addition to the cytokine-dependent iNOS activation, also non-immune iNOS activation has been observed in hRPE cells. Here, iNOS was induced by all-trans retinal, an intermediate in the vision cycle, or by cultivation under high-glucose conditions [22, 38]. In addition, iNOS is detectable in RPE cells from other species, including pigs, cows, mice, and rats [18, 39]. Interestingly, iNOS has potent antimicrobial activities and inhibits the spread of murine CMV, but overexpression of iNOS can also damage RPE cells, change neovascularization in ischemic retinopathy, and is associated with the severity of diabetic retinopathy in mice and humans [16, 19, 40].

Here, we found that hRPE cells do not express iNOS activity after IFN-γ stimulation, but after co-stimulation of hRPE cells with IFN-γ/IL-1β as well as IFN-γ/TNF-α/IL-1β. The magnitude of nitric oxide production is dependent on several factors, including cell number, cytokine concentration, and cultivation time. iNOS activity in hRPE cells could be blocked by addition of the NOS antagonist *N*^G^-monomethyl-l-arginine (N^G^MMA). We confirmed via real-time PCR analyses that the inducible form of NO-synthases (iNOS, also known as NOS2) was indeed upregulated after co-stimulation with IFN-γ, TNFα, and IL-1β, whereas no noteworthy mRNA amounts of endothelial (eNOS also known as NOS 3) or neuronal NO-synthases (nNOS, also known as NOS 1) were detectable. IDO1 as well as iNOS are described as potent antimicrobial effector mechanisms [41]. However, we could not find a synergistic antimicrobial effect of both IFN-γ-induced effector mechanisms directed against *S. aureus* or *T. gondii*. Instead, iNOS activity generated sufficient NO concentrations that were capable to inhibit IDO1 activity, which could be reversed by iNOS inhibition via N^G^MMA administration. Several possibilities such as altered transcription, translation, or post-translational modifications could account for this NO-mediated IDO1 inhibition. Relative IDO1 mRNA expression levels, as detected by real-time PCR analyses, were not influenced by the presence of NO; hence, a reduced IDO1 transcription cannot be responsible for the low IDO1 activity. Furthermore, IDO1 protein levels are unaffected in different single or combinatorial cytokine stimulation assays as shown by western blot analyses. Due to that, we expect that NO does not influence IDO1 on a translational level in hRPE. In conclusion, we assume that NO directly interacts with the IDO1 protein. Such an interaction was published by Thomas et al. before [42]. They described the direct interaction of NO with the heme group of the IDO1 molecule in a cell-free environment. To elucidate the IDO1-NO interaction in hRPE cells in more detail, it would be necessary to purify IDO1 protein for biochemical studies using a NO donor.

In a previous publication, we described a comparable direct effect of iNOS on IDO enzyme activity using lysates of activated RT4 cells (human uroepithelial carcinoma cell line) and a chemical NO donor [37]. In addition, we have previously described that iNOS activation causes an enhanced proteasomal degradation of IDO1 protein in RT4 cells [37]. A comparable iNOS-dependent loss of IDO protein expression in hepatocytes was described by Bando et al. very recently [43]. However, it is not very likely that the proteasomal degradation mechanism takes place in hRPE cells, since we did not find a reduced IDO protein expression in IFN-γ, TNFα, and IL-1β single or co-stimulated hRPE cells. In addition, there was no reduction of IDO1 mRNA detectable in iNOS-positive cells.

Here, we show that iNOS-mediated inhibition of IDO1 in hRPE cells abrogates also subsequent antiparasitic and antibacterial effects. In functional analyses, we found that the iNOS inhibitor N^G^MMA restored the capacity of hRPE cells to inhibit *S. aureus* and *T. gondii* growth. This opens the question which function the iNOS-mediated IDO1 inhibition might have in human cells. In general, iNOS production by human cells in vitro is not intense and in many cases undetectable. In contrast, murine cells often produce high amounts of nitric oxide, which are sufficient to inhibit the growth of pathogens, for example, *T. gondii* [44]. In addition, other IFN-γ-induced antimicrobial effector mechanisms, such as immunity-related guanosine triphosphatases and guanylate binding proteins, act in concert with iNOS to inhibit *T. gondii* growth in murine cells. In contrast, in human cells, IDO1 is an important antimicrobial protein. The intensity of IDO induction directly correlates with the amount of IFN-γ used for stimulation. Since IDO is the rate-limiting enzyme in the kynurenine pathway, an excessive IDO1 induction results in tryptophan starvation, which is an unfavorable situation for the host cells. Furthermore, the production of toxic metabolites along the kynurenine pathway such as kynurenine and 3OH-kynurenine might be harmful for immune and tissue cells [45, 46] or induce a plethora of effects via aryl hydrocarbon receptor signaling [47]. Therefore, control of IDO1 activity by NO might be beneficial to reduce detrimental IDO1-mediated cell damage.

## Electronic supplementary material

Below is the link to the electronic supplementary material.

Supplementary material 1 (DOCX 210 kb)
