# Supplementary material for: Interplay between IDO1 and iNOS in human retinal pigment epithelial cells
Source: Med Microbiol Immunol. 2019 Jul 2;208(6):811–24. doi: 10.1007/s00430-019-00627-4 (PMC6817751; doi:10.1007/s00430-019-00627-4)
Supplement: Supplementary file 1 — Supplementary material 1 (DOCX 210 kb) [file 430_2019_627_MOESM1_ESM.docx]

**Interplay between IDO1 and iNOS in human retinal pigment epithelial cells**

Katrin Spekker-Bosker*, Christoph-Martin Ufermann*, Maike Oldenburg, Walter Däubener and Silvia Kathrin Eller^§^

Institute of Medical Microbiology and Hospital Hygiene, Heinrich-Heine-University, Düsseldorf, Germany

* These authors contributed equally to this work.

^§^corresponding author:

Dr. Silvia Kathrin Eller

Institute of Medical Microbiology and Hospital Hygiene

Heinrich-Heine-University Düsseldorf

Universitätsstr. 1, Bldg. 22.21

40225 Düsseldorf, Germany

e-mail: [silvia.eller@uni-duesseldorf.de](mailto:silvia.eller@uni-duesseldorf.de)

**Content Page**

**Electronic Supplementary Material 1: 1-Methyl-L-Tryptophan (1-MT) treatment and**

**hypoxic conditions (1 % oxygen) abrogate indoleamine 2,3-dioxygenase (IDO) - mediated antimicrobial effects in human retinal pigment epithelial (hRPE) cells ESM-2**

**
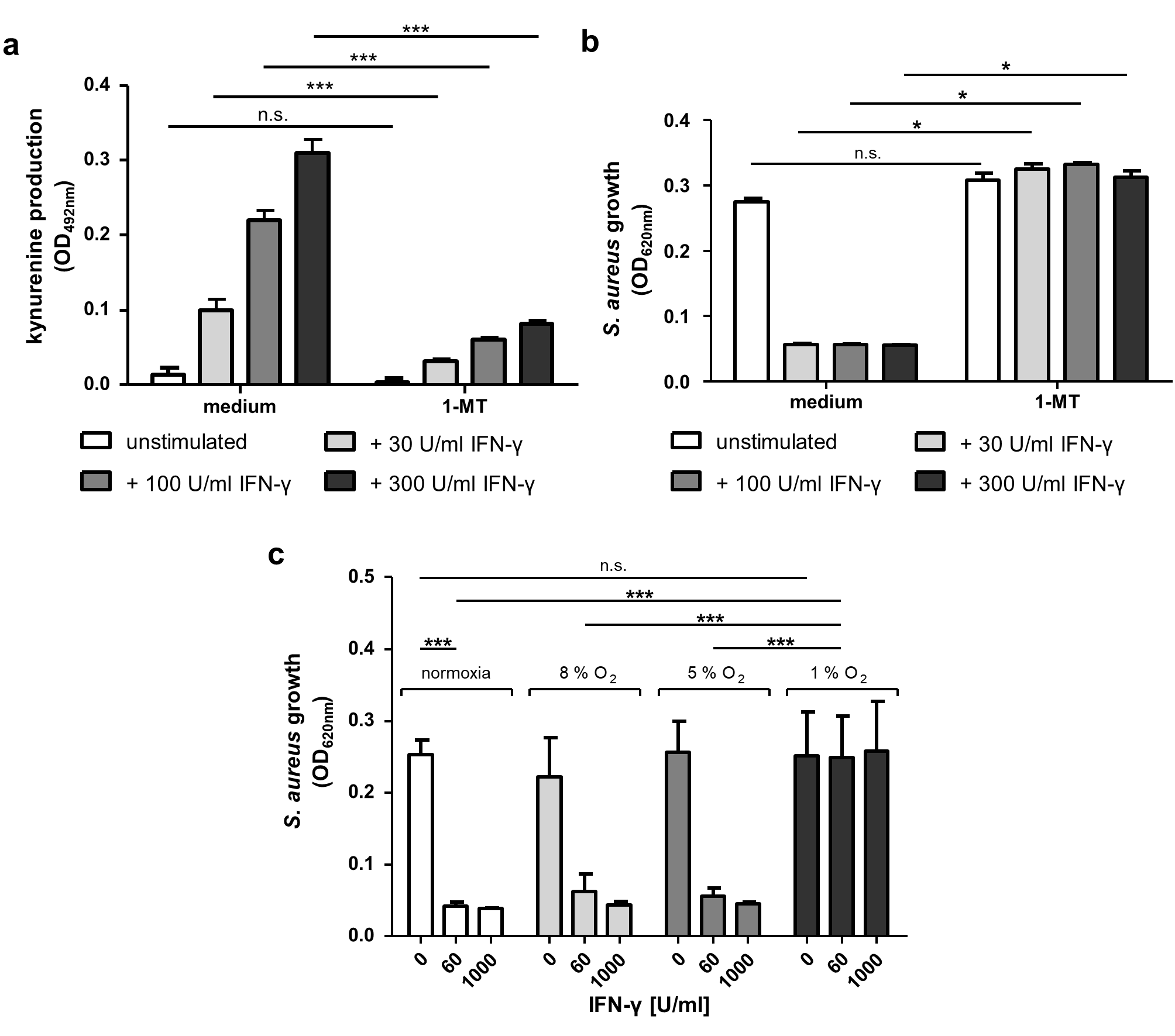
**

**Electronic Supplementary Material 1: 1-Methyl-L-Tryptophan (1-MT) treatment and hypoxic conditions (1 % oxygen) abrogate indoleamine 2,3-dioxygenase (IDO) - mediated antimicrobial effects in human retinal pigment epithelial (hRPE) cells.** (a) 3 x 10^4^ hRPE cells were left unstimulated or stimulated in 96-well plates with indicated amounts of human IFN-γ (30 U/ml, 100 U/ml or 300 U/ml) and/or additionally treated with 1-MT (1.5 mM) in the presence of L‑tryptophan (100 µg/ml). After 72 h the cell culture supernatants were harvested and the kynurenine content was determined by use of Ehrlich´s reagent. (b) IFN-γ and/or 1-MT (1.5 mM) pre-stimulated hRPE cells (for 72 h) were infected with *Staphylococcus aureus* (10-100 cfu/well). The bacterial growth was detected by measurement of the optical density at 620 nm (OD_620nm_) after additional 16 h. (c) 5 x 10^5^ hRPE cells were stimulated in 24-well plates with indicated IFN-γ concentrations under atmospheric oxygen concentration (normoxic) or hypoxic (8 % O_2_, 5 % O_2_ or 1 % O_2_) conditions (for 72 h). Cell culture supernatants were inoculated with *S. aureus* and bacterial growth was detected by measurement of the optical density at 620 nm (OD_620nm_) after 16 h. Data are given as mean ± SEM of three experiments, each performed in triplicate. Significant differences are indicated with asterisks (n.s. = not significant; ∗ p ≤ 0.05; ∗∗ p ≤ 0.001 and ∗∗∗ p ≤ 0.0001). The unpaired, two-tailed student´s t test was used.
